# Whole grain intake and its association with intakes of other foods, nutrients and markers of health in the National Diet and Nutrition Survey rolling programme 2008–11

**DOI:** 10.1017/S0007114515000525

**Published:** 2015-05-28

**Authors:** Kay D. Mann, Mark S. Pearce, Brigid McKevith, Frank Thielecke, Chris J. Seal

**Affiliations:** 1 Institute of Health and Society, Newcastle University, Newcastle upon TyneNE1 4LP, UK; 2 Human Nutrition Research Centre, School of Agriculture, Food and Rural Development, Agriculture Building, Kings Road, Newcastle University, Newcastle upon TyneNE1 7RU, UK; 3 Cereal Partners UK, Welwyn Garden CityAL7 1RR, UK; 4 Cereal Partners Worldwide, Lausanne, Switzerland; 5 Nestlé Research Center, Vers chez les Blanc, Lausanne, Switzerland

**Keywords:** Whole grain intake, Whole grain associations, UK population, UK diet

## Abstract

Epidemiological evidence suggests an inverse association between whole grain consumption and the risk of non-communicable diseases, such as CVD, type 2 diabetes, obesity and some cancers. A recent analysis of the National Diet and Nutrition Survey rolling programme (NDNS-RP) has shown lower intake of whole grain in the UK. It is important to understand whether the health benefits associated with whole grain intake are present at low levels of consumption. The present study aimed to investigate the association of whole grain intake with intakes of other foods, nutrients and markers of health (anthropometric and blood measures) in the NDNS-RP 2008–11, a representative dietary survey of UK households. A 4-d diet diary was completed by 3073 individuals. Anthropometric measures, blood pressure levels, and blood and urine samples were collected after diary completion. Individual whole grain intake was calculated with consumers categorised into tertiles of intake. Higher intake of whole grain was associated with significantly decreased leucocyte counts. Significantly higher concentrations of C-reactive protein were seen in adults in the lowest tertile of whole grain intake. No associations with the remaining health markers were seen, after adjustments for sex and age. Over 70 % of this population did not consume the minimum recommend intake associated with disease risk reduction, which may explain small variation across health markers. Nutrient intakes in consumers compared with non-consumers were closer to dietary reference values, such as higher intakes of fibre, Mg and Fe, and lower intakes of Na, suggesting that higher intake of whole grain is associated with improved diet quality.

Whole grains and foods containing whole grains, such as whole-grain breads, porridge and ready-to-eat cereals, are considered to be beneficial to health. Epidemiological evidence suggests an inverse linear association between whole grain consumption and the risk of many non-communicable diseases, such as CVD, type 2 diabetes (T2D), obesity and some cancers^(^ ^1^ ^,^ ^2^ ^)^. Observational studies have shown that those consuming 2–3 servings of whole grains per d (the upper level in most studies) have around a 21 % reduction in the risk of CVD and around a 26 % reduction in the risk of T2D compared with never or rare consumers^(^ ^1^ ^,^ ^3^ ^)^. However, intervention studies have shown no such reduction in the risk of CVD markers in whole grain consumers over a 4-month period^(^ ^4^ ^)^. Consumers of whole grain have also been shown to have a better overall diet quality, particularly for increased fibre intake^(^ ^5^ ^)^. Along with insoluble fibre, bioactive components found in bran and germ of whole grains, such as antioxidant micronutrients, carotenoids, minerals, polyphenols and vitamins (to name a few), are attributed to the protective effects of whole grains on health^(^ ^6^ ^)^.

The recommendation to increase whole grain consumption is now global; however, dietary and quantity-specific guidance across countries is variable, with no quantitative recommendation in the UK^(^ ^3^ ^)^. A recent analysis of the UK dietary data has shown that the average whole grain intake is low in both adults (20 g/d) and children (13 g/d)^(^ ^7^ ^)^, much lower than the suggested 2–3 daily servings (32–48 g/d) recommended by the US Department of Agriculture (USDA) in the 2010 Dietary Guidelines for Americans^(^ ^8^ ^)^, and the recommendation of 75 g whole grain/10 MJ from the Danish Nutrition Foundation^(^ ^9^ ^)^. It is important to understand whether the health benefits associated with whole grain intake previously attributed to higher levels of intake are still present at these low levels of consumption seen in the UK.

The aim of the present study was to investigate the association of whole grain intake with intakes of other foods, nutrients and markers of health (anthropometric and blood measures) in the National Diet and Nutrition Survey rolling programme (NDNS-RP) 2008–11.

## Methods

### Study population

The NDNS-RP assesses the diet, nutritional intake and status of individuals aged 1·5 years and above, living in private households in the UK (England, Scotland, Wales and Northern Ireland). Between April 2008 and March 2011, 3073 randomly selected adults and children were invited to complete a 4-d food diary, interviewed and asked to provide blood and urine samples. Further details of the NDNS-RP methodology have been described elsewhere^(^ ^10^ ^,^ ^11^ ^)^. In brief, participants completed an estimated food diary recording all foods and drinks consumed both at home and away from home for four and, in some cases (*n* 53, 2 %), three consecutive days. Participants also completed a computer-assisted personal interview, collecting information on dietary habits and lifestyle, and had height and weight measured. In a follow-up nurse visit to the household, waist:hip ratio and blood pressure were measured, and fasting blood samples were taken for those aged 4 years and above.

Food diaries for those aged 11 years and younger were completed by a parent/carer with help from the child. Processing of the food diary data was done by trained coders and editors. Food intakes were entered into the MRC HNR's (Medical Research Council, Human Nutrition Research) dietary assessment system, Diet In Nutrients Out^(^ ^12^ ^)^. The food composition data used was from the Department of Health's NDNS Nutrient Databank. Data coders matched each food/drink item recorded in the diary with a food code and portion code from Diet In Nutrients Out. For composite items (e.g. sandwiches) and home-made meals, their component parts were assigned individual food codes. Further details of data coding and editing are outlined in Appendix A of the NDNS-RP official report^(^ ^13^ ^)^. Food intake groups included milk, cheese, yogurt, eggs, fats, meat, fish, fruit, vegetables, pasta, rice, bread, ready-to-eat cereals, biscuits, buns, cakes, pastries, confectioneries, savoury crisps and snacks. Average daily food and nutrient intakes were derived from the food diary data by the NDNS-RP team.

Height and weight were measured at the first participant visit using a portable stadiometer and weighing scales. BMI was calculated, and for participants whose height could not be measured, estimated height based on demispan (the distance between the mid-point of the sternal notch and the finger roots with the arm outstretched laterally) was used to calculate the BMI^(^ ^14^ ^)^. Waist and hip circumferences were measured during a nurse visit to the participants aged 11 years and above using a tape measure. All measurements were taken twice and a third was taken if there was a discrepancy between the first two measurements above a given value (height ≥ 0·5 cm, weight ≥ 0·2 kg, waist and hip circumferences ≥ 3 cm). For the purpose of this analysis, the mean of the closest two measurements was used.

Blood pressure level, collected during the nurse visit to the participant, was measured in the sitting position using an automated, validated machine (Omron HEM907), after 5 min of rest. Blood pressure level was only collected for participants aged 4 years and above. The mean of the second and third blood pressure readings taken at 1 min intervals in participants who had not eaten, drunk alcohol, exercised or smoked in the preceding 30 min was used. Individuals who did not have three valid blood pressure readings were excluded from the analysis.

Overnight fasting blood samples were obtained from those aged 4 years and above and from non-diabetics, otherwise non-fasting samples were obtained. However, for the purpose of this analysis, only blood analytes from participants aged 11 years and above have been investigated due to small numbers (19 %) of participants aged 1·5–10 years providing blood samples. We included data from 13/37 adults with diabetes who provided non-fasting samples and for whom analyte data were available. Blood samples were analysed for a range of analytes^(^ ^10^ ^)^, including Hb, serum total cholesterol, HDL-cholesterol, LDL-cholesterol, C-reactive protein (CRP), TAG, plasma creatinine and leucocyte counts.

The NDNS-RP was conducted according to the guidelines laid down in the Declaration of Helsinki, and ethical approval for all procedures was granted by Local Research Ethics Committees covering all areas covered in the survey. All participants gave informed consent.

### Estimation of whole grain intake

As has been described previously^(^ ^7^ ^)^, food diary data were investigated for foods containing any whole-grain ingredient (*n* 221), using the HEALTHGRAIN definition of whole grain^(^ ^15^ ^)^ and classification of whole-grain ingredients outlined in the study by Seal and colleagues^(^ ^16^ ^)^. Whole-grain content of each food identified was calculated per 100 g, on a DM as consumed basis, and absolute daily whole grain intake in grams was calculated for consumers along with tertiles of intake. A non-consumer of whole grain was defined as an individual who did not consume any foods containing any amount of whole-grain ingredient.

### Weighting the data

Data used in the analysis were weighted in order to exclude any potential selection bias in the observed results arising from non-response bias in the NDNS-RP. Weighting variables to account for any potential bias in households, main food provider, individual selection, seasonality and for age, sex and regional profiles of participating individuals was provided by the NDNS team. Weighting for non-response to nurse visit and providing a blood sample were also used in the respective analyses. Full details of weight computation are described in Appendix B of the NDNS national report^(^ ^17^ ^)^.

### Statistical analysis

Means and standard deviations of food and nutrient intakes were calculated for non-consumers of whole grain and by tertile of intake for consumers. Means and standard deviations of health marker measures were also calculated for non-consumers of whole grain and by tertile of intake for consumers. Associations across whole grain intakes (non-consumers and tertile groups) and intakes of other foods, nutrients and health markers were investigated using linear regression with values adjusted for sex and age in multivariable models; the dependent variable was the outcome of interest (e.g. fibre intake) and the independent predictor was intake group (non-consumer, tertile 1, tertile 2 and tertile 3). To eliminate potential confounding by sex and age, any significant associations were then adjusted for sex and, if the association remained significant, further adjusted for age. Differences between non-consumers and tertiles of whole grain intake were assessed using independent *t* tests, adjusting for sex, within the regression modelling. *P*< 0·05 was used to denote significance throughout all statistical analysis.

All statistical analysis was performed in Stata version 12 (StataCorp) utilising the complex survey functions.

## Results

In this population of 1052 children/teenagers (1·5–17 years) and 1521 adults (18+ years), 15 % of children/teenagers and 18 % of adults did not consume any whole grain (Table 1). The mean whole grain intake ranged from 5·4 (sd 2·8) g/d in tertile 1 to 40·1 (sd16·4) g/d in tertile 3 for children/teenagers and from 8·4 (sd 4·6) g/d in tertile 1 to 61 (sd 26·8) g/d in tertile 3 for adults. The mean whole grain intake of consumers included only in tertile 3 (29 % of children/teenagers, 28 % of adults and 29 % of the total population) achieved the minimum recommendation (32 g/d) for reduced risk of non-communicable disease. The percentages of males and females across non-consumers and tertile groups were similar in children/teenagers and adults, although there was a higher proportion of child/teenage males in tertile 3 (Table 1). Mean age was lower across non-consumers and tertile groups in children/teenagers and was greater across adult non-consumers and tertile groups. Age differences in whole grain intake have been reported elsewhere^(^ ^7^ ^)^.

**Table 1:** Whole grain intake in children/teenagers and in adults (Mean values and standard deviations, and numbers and percentages of participants)

	Whole grain intake (g/d)
	Non-consumers	T1 (lowest)	T2	T3 (highest)	Total population
	Mean	sd	Mean	sd	Mean	sd	Mean	sd	Mean	sd
Children/teenagers (1·5–17 years)	0		5·4	2·8	16·5	3·7	40·1	16·4	17·8	18·1
Sex (% male)	50	44	49	60	51
Age (years)	10·7	5·2	9·6	4·9	9·1	4·7	8·9	4·7	9·4	4·9
Unweighted										
*n*	227	415	418	442	1502
%	15	28	28	29	100
Adults (18+ years)	0		8·4	4·6	25·7	5·5	61·0	26·8	26·2	27·8
Sex (% male)	56	44	43	55	49
Age (years)	40·6	17·7	44·1	19·1	49·6	18·9	49·4	17·7	46·4	18·8
Unweighted										
*n*	277	431	426	437	1571
%	18	27	27	28	100

T, tertile.

### Intakes of nutrients

Whole grain consumers had significantly different intakes of some nutrients than non-consumers (Table 2). Nutrient intakes reported here come exclusively from foods consumed over the diet diary and do not include vitamin or mineral supplements. Significant associations for higher intakes of whole grain were seen with greater intake of total energy and energy from protein, carbohydrates and total sugar (in children/teenagers only). Higher intake of whole grain was also significantly associated with greater intake of fibre, Fe, Ca, vitamin E (in adults only), K, P, Mg, thiamin, riboflavin, niacin (in children/teenagers only), vitamin B12 (in adults only) and vitamin D (in adults only). Higher intake of whole grain was significantly associated with lower intake of energy from non-milk extrinsic sugar, fat and alcohol (in adults only).

**Table 2:** Mean nutrient intakes of non-consumers and by tertiles (T) of whole grain intake

	Child/teenage mean		Adult mean	
	Whole grain intake/d		Whole grain intake/d	
Nutrient	0 g/d	T1	T2	T3	*P* †	0 g/d	T1	T2	T3	*P* †
Energy (kcal)	1549	1528	1582	1683*	< 0·001	1862	1743	1784	1987*	< 0·001
Energy (MJ)	6·5	6·4	6·7	7·1*	< 0·001	7·8	7·3	7·5	8·4*	< 0·001
% energy from protein	14·8	14·6	14·5	15·1	< 0·05	15·8	16·2	16·9*	16·7*	< 0·01
% energy from carbohydrate	50·3	50·7	51·5*	51·8*	< 0·01	44·9	44·5	45·5	46·6*	< 0·001
% energy from total sugar	21·8	22·7	23·6*	23·0*	< 0·01	19·5	19·1	19·8	19·9	≥ 0·05
% energy from NME sugars	14·3	14·6	14·8	13·8	< 0·01	13·6	12*	11·2*	10·6*	< 0·001
% energy from fat	34·7	34·4	33·8*	33·0*	< 0·001	33·7	34·0	32·9	32·8	< 0·05
% energy from saturated fat	13·0	13·4	13·3	13·1	≥ 0·05	12·4	12·7	12·2	12·2	≥ 0·05
% energy from alcohol	0·2	0·2	0·1	0·1	≥ 0·05	5·6	5·3	4·7	3·9*	< 0·05
Fibre (g/10 MJ)	14·8	15·1*	16·8*	19·3*	< 0·001	14·4	16·2*	19·2*	21·6*	< 0·001
Na (mg/10 MJ)	3051	2978	2885*	2910*	≥ 0·05	3090	3027	2989	2934*	≥ 0·05
Fe (mg/10 MJ)	11·7	12·3*	13·3*	15·0*	< 0·001	12·3	13·6*	14·7*	15·2*	< 0·001
Ca (mg/10 MJ)	1147	1169	1234*	1276*	< 0·001	984	1044	1114*	1149*	< 0·001
Vitamin E (mg/10 MJ)	11·1	10·5*	10·7	10·5	≥ 0·05	11·0	11·2	11·8	12·1*	< 0·001
K (mg/10 MJ)	3233	3255	3322	3422*	< 0·001	3303	3549*	3884*	3916*	< 0·001
P (mg/10 MJ)	1489	1520	1563*	1665*	< 0·001	1502	1556	1674*	1752*	< 0·001
Mg (mg/10 MJ)	264·7	276·1*	294·3*	326*	< 0·001	286	306*	344*	376*	< 0·001
Thiamin (mg/10 MJ)	1·8	1·9*	2·0*	2·1*	< 0·001	1·8	1·8	2·0*	2·0*	< 0·001
Riboflavin (mg/10 MJ)	2·0	2·1	2·3*	2·4*	< 0·001	1·8	2·0	2·2*	2·3*	< 0·001
Niacin (mg/10 MJ)	41·7	41·7	41·4	44·2*	< 0·01	47·9	46·7	48·5	49·1	≥ 0·05
Vitamin B6 (mg/10 MJ)	2·8	2·8	2·8	2·9	≥ 0·05	3·1	2·9	3·0	3·0	≥ 0·05
Vitamin B12 (μg/10 MJ)	6·0	5·8	6·1	6·4	≥ 0·05	6·7	6·9	7·6	7·7*	< 0·05
Vitamin D (μg/10 MJ)	3·3	2·9	3·1	3·1	≥ 0·05	3·7	3·5	4·0	4·1*	< 0·01
*n* (unweighted)	227	415	418	442		277	431	426	437	

NME, non-milk extrinsic.

* Values are significantly different from non-consumers (*P*< 0·05; *t* test, adjusted for sex).

† Association across intakes of whole grain and nutrient intakes (linear regression, adjusted for sex).

Compared with non-consumers of whole-grain foods, consumers in tertile 3 had significantly higher intakes of total energy, energy from protein (in adults only), carbohydrates and total sugar (in children/teenagers only) and significantly higher intakes of fibre, Fe, Ca, vitamin E (in adults only), K, P, Mg, thiamin, riboflavin, niacin (in children/teenagers only), vitamin B12 (in adults only) and vitamin D (in adults only) (Table 2). Consumers in tertile 3 also had significantly lower intakes of energy from non-milk extrinsic sugar (in adults only), fat (in children/teenagers only) and alcohol (in adults only), and significantly lower intakes of Na compared with non-consumers of whole-grain foods.

After adjustment for sex and age, the associations of higher intake of whole grain with greater intakes of vitamins B12 and D in adults only were no longer significant. Adjustment for sex and age did not attenuate any other of the associations found with nutrient intakes.

### Intakes of other foods

Higher intake of whole grain was significantly associated with greater intake of milk, cheese (in children/teenagers only), yogurt, fats and spreads, fish (in adults only), fruit and vegetables, wholemeal bread, ready-to-eat cereals, biscuits and cakes (in adults only) (Table 3). Higher intake of whole grain was also significantly associated with lower intake of white meat (in children/teenagers only), red meat (in adults only), white bread and savoury crisps and snacks (in adults only).

**Table 3:** Mean intakes of other foods in non-consumers and by tertiles (T) of whole grain intake

	Child/teenage mean		Adult mean	
	Whole grain intake/d		Whole grain intake/d	
*Food (g)*	0 g/d	T1	T2	T3	*P* †	0 g/d	T1	T2	T3	*P* †
Milk	184·8	177·2	207·2	237·1*	< 0·01	117·4	135·8*	170*	197·6*	< 0·001
Cheese	8·2	10·3	9·6	11·7*	< 0·01	14	13·2	14·2	17·5	≥ 0·05
Yogurt	18·1	25·6*	31·8*	41·1*	< 0·001	13·8	26·5*	27·4*	41*	< 0·001
Eggs and egg dishes	12·9	9·0*	11·0	11·0	≥ 0·05	18·4	17·1	18·8	20·9	≥ 0·05
Fats and spreads	8·6	7·9	7·7	9·5	< 0·01	12·2	10·4	10·0*	13·4	< 0·001
Meat	85·3	78·9	74·2*	75·8*	≥ 0·05	121·3	108·1	99·2*	98·9*	< 0·01
White meat	32·6	28	25·6*	23·5*	< 0·01	37·8	36·6	33·8	33·7	≥ 0·05
Red meat	52·7	51	48·6	52·2	≥ 0·05	83·5	71·6	65·4*	65·2*	< 0·001
Fish and fish dishes	9·3	9·8	10·4	11·9	≥ 0·05	15·2	19·5*	27·5*	30·4*	< 0·001
Fruit and vegetables	154·6	173·5*	193·1*	220·2*	< 0·001	214·1	245*	302·3*	352·8*	< 0·001
Fruit	63·2	79·9*	92*	105·9*	< 0·001	60·3	83·7*	110·4*	142*	< 0·001
Vegetables	91·4	93·6	101·1	114·3*	< 0·001	153·9	161·3	191·9*	210·8*	< 0·001
Pasta, rice and other cereals	87·5	76·8	76·8	79·1	≥ 0·05	74·1	72·9	69·4	81·7	≥ 0·05
Bread (all breads)	68·7	65·1	68·8	82·3*	< 0·001	89·1	73·6*	76*	99·6*	< 0·001
White bread	62·3	55·1	48·2*	34·2*	< 0·001	82	56·5*	42·2*	27·6*	< 0·001
Wholemeal bread	0	1·1*	5·1*	22*	< 0·001	0	2·9*	13·2*	48·7*	< 0·001
RTEC	9·5	18·8*	26·5*	39·9*	< 0·001	5·32	16·7*	33·9*	46·3*	< 0·001
High-fibre RTEC	0	7·4*	17*	30·8*	< 0·001	0·19	9·2*	28*	40·7	< 0·001
Other RTEC	9·5	11·4	9·5	9·1	≥ 0·05	5·1	7·5*	5·9	5·6	≥ 0·05
Biscuits	11·6	14·1	17·1*	17·1*	< 0·01	8·0	11·6*	13·5*	17·1*	< 0·001
Buns, cakes, pastries and pies	15·5	18·1	18·9	18·6	≥ 0·05	14·6	17·8	21·4*	23·3*	< 0·01
Confectioneries	15·3	19·5	17·3	17·8	≥ 0·05	10·8	10·2	8·9	9·5	≥ 0·05
Sugar confectionery	4·8	9·2*	7·7*	7·5*	< 0·05	1·2	2·4	1·4	1·7	≥ 0·05
Chocolate confectionery	10·5	10·2	9·6	10·3	≥ 0·05	9·6	7·8	7·5	7·8	≥ 0·05
Savoury crisps and snacks	12·4	10·1	11·7	10·3	≥ 0·05	8·2	6·4	6·1*	5·3*	< 0·05
*n* (unweighted)	227	415	418	442		277	431	426	437	

RTEC, ready-to-eat cereals.

* Values are significantly different from non-consumers (*P*< 0·05; *t* test, adjusted for sex).

† Association across intakes of whole grain and other food intakes (linear regression, adjusted for sex).

Mean intakes of milk, cheese (in children/teenagers only), yogurt, fish (in adults only), fruit and vegetables, wholemeal bread, ready-to-eat cereals, biscuits, cakes (in adults only) and sugar confectioneries (in children/teenagers only) were significantly higher in consumers in tertile 3 compared with non-consumers (Table 3). However, mean intakes of white meat (in children/teenagers only), red meat (in adults only), white bread, and savoury crisps and snacks (in adults only) were significantly lower in consumers in tertile 3 compared with non-consumers. Adjustment for age and sex did not attenuate any of the associations found with intakes of other foods.

### Health markers

Significant associations were seen from lowest to highest levels of whole grain intake with a fall in systolic blood pressure (in children/teenagers only) and a fall in leucocyte counts (Table 4). After adjustment for age, the association with systolic blood pressure in children/teenagers was no longer significant. In comparison with non-consumers of whole grain, adult consumers in tertile 1 had significantly lower CRP concentrations (tertile 1: 2·8 *v.* 3·8 mg/l; *P*= 0·04). However, the reductions in CRP concentrations of adult consumers in tertile 2 and tertile 3, although present, were not significantly different from non-consumers (Table 4). Child/teenage consumers in tertile 2 and tertile 3 had significant lower systolic blood pressure than non-consumers (tertile 2: 107·1, tertile 3: 107·4 *v.* 110·3 mmHg, tertile 2: *P*= 0·02, tertile 3: *P*= 0·01); however, this association did not remain after adjustment for age. No other significant associations or differences were seen between whole grain intake and other health markers.

**Table 4:** Mean health marker outcome for non-consumers and by tertiles (T) of whole grain intake

	Child/teenage mean		Adult mean	
	Whole grain intake/d		Whole grain intake/d	
*Health marker*	*0* *g/d*	*T1*	*T2*	*T3*	P	*0* *g/d*	*T1*	*T2*	*T3*	P
Weight (kg)	60·1	57·8	56	57·4	0·21	78·1	76·8	76·8	77·9	0·91
BMI	22	21·9	21·3	21·1	0·38	27·2	27·3	27·3	27·1	0·94
*n* (unweighted)	120	155	146	147		240	401	394	408	
Waist:hip ratio	0·8	0·8	0·8	0·8	0·78	0·9	0·9	0·9	0·9	0·14
*n* (unweighted)	94	119	112	104		199	316	329	312	
Systolic blood pressure (mmHg)	110·3	109·3	107·1*	107·4*	0·03†	125·0	126·4	127·3	127·7	0·48
Diastolic blood pressure (mmHg)	63·3	63·3	62·6	61·6	0·36	73·0	73·3	73·3	73·9	0·94
*n* (unweighted)	111	192	193	186		138	256	272	254	
Cholesterol (mmol/l)	3·9	4·2	4·0	4·0	0·45	5·1	5·2	5·1	5·2	0·57
HDL (mmol/l)	1·4	1·4	1·4	1·4	0·80	1·4	1·5	1·5	1·5	0·45
LDL (mmol/l)	2·2	2·5	2·3	2·3	0·28	3·1	3·1	3·0	3·2	0·38
C-reactive protein (mg/l)	3·0	2·1	2·0	1·6	0·29	3·8	2·8*	2·9	3·0	0·23
TAG (mmol/l)	0·8	0·8	0·8	0·7	0·74	1·5	1·4	1·3	1·3	0·36
Leucocyte count (109/l)	7·3	5·9	6·2	6·0	0·04†	6·9	6·5	6·3*	5·9*	0·01†
Hb (g/dl)	141	136	135	138	0·22	143	140	141	142	0·59
Creatinine (μmol/l)	66·1	65·9	62·4	16·8	0·43	79·6	79·7	80·8	82·9	0·46
*n* (unweighted)	42	51	54	53		87	160	163	170	

* Values are significantly different from non-consumers (*P*< 0·05; *t* test, adjusted for sex).

† *P*< 0·05 association across intakes of whole grain and health marker (linear regression, adjusted for sex).

## Discussion

In this UK population, with low consumption of whole grain^(^ ^7^ ^)^, a significant decrease in leucocyte counts was seen across groups of intake, after adjustment for age and sex. In adults, CRP concentrations were significantly lower in low consumers compared with non-consumers. However, no other significant associations were seen in either adults or children/teenagers with other markers of health. Intakes of other foods and nutrients in whole grain consumers were closer to dietary reference values and indicative of an overall better diet.

We have shown in this population that the diets of whole grain consumers was more nutrient dense than that of non-consumers. Intakes of a number of vitamins and minerals (e.g. Fe and magnesium and several B vitamins) were significantly increased in those consuming whole grain. This reflects the naturally higher content of these nutrients in whole-grain foods that are lost in the manufacture of refined flours unless replaced by mandatory fortification. It is difficult to rule out confounding of overall healthy diet and lifestyle that comes hand in hand with whole grain consumption, so it is not clear whether these differences in nutrient intake are solely due to the intake of whole grain or the combined effect of consuming a healthier diet overall. However, this pattern of improved nutrient profile has also seen in similar population studies investigating the difference between whole grain consumers and non-consumers in North America and Europe ^(18–22)^.

For many populations, including the US population, whole-grain foods make a substantial contribution to total dietary fibre intake^(^ ^5^ ^)^. Our findings, for this cross-section of the UK population, confirm this observation, with nutritionally significant higher fibre intakes in those that consume even a small amount of whole grain (on average 5 g/d more in children/teenagers and 8 g/d more in adults). It is important to note that the higher dietary fibre intake reported here may not exclusively come from whole-grain foods, but may also come from other sources such as fruit and vegetables, intakes of which were significantly higher in both children/teenager and adult whole grain consumers compared with non-consumers (Table 3). However, the recommended intake of five portions of fruit and vegetables per d was not achieved by 25 % of the study population^(^ ^10^ ^)^. Most whole-grain cereals are a rich source of fibre; for example, whole-grain wheat contains between 9 and 17 g of fibre per 100 g, more than most vegetables (generally < 6 g/100 g)^(^ ^6^ ^)^. This suggests that the majority of the increase in fibre intake may be attributed to the increase in whole grain intake. On the basis of the epidemiological evidence of the benefits of increased whole grain and fibre intakes, the recently revised Nordic nutrition recommendations^(^ ^23^ ^)^ now state that fibre intake should come from ‘foods naturally rich in dietary fibre such as whole grain…’ whereas whole grains had not been mentioned before. A greater emphasis on a recommendation to increase whole grain intake in the UK would potentially contribute to increasing fibre intakes in this population, as seen in a 4-month whole-grain food intervention study where a marked increase in dietary fibre intake was achieved compared with baseline^(^ ^4^ ^)^. Putting greater emphasis on increasing whole grain consumption will be essential if the recommendation by the UK Scientific Advisory Committee on Nutrition to increase the dietary reference value for dietary fibre for an adult population to 30 g/d is to be achieved^(^ ^24^ ^)^.

In addition to the fibre contained in whole grains, there are also fatty acids, oligosaccharides and antioxidants that have anti-inflammatory and immune stimulating properties in the human body^(^ ^6^ ^)^. Across tertiles of whole grain intake, we observed a significant reduction in leucocyte counts and a difference in adult CRP concentrations between non-consumers and low whole grain consumers. Leucocyte counts and CRP concentrations are markers of immune response and inflammation within the body, and are linked to the pathogenesis of CVD and T2D^(^ ^25^ ^,^ ^26^ ^)^. It is interesting that even at the reported low level of whole grain intake in this population, such associations were observed. Similar changes in CRP were seen in two cross-sectional studies where CRP concentrations were lower in whole-grain bread consumers^(^ ^27^ ^)^ and across whole grain intake categories^(^ ^28^ ^)^. In contrast to the results reported here, the associations of whole grain intake with inflammatory markers are not consistently supported by intervention studies^(^ ^29^ ^)^. Many of these studies have reported no change in inflammatory markers, notably CRP and IL-6 over 4-month, 12- and 6-week intervention periods^(^ ^4^ ^,^ ^30^ ^,^ ^31^ ^)^. The difference in observation may be due to short-term dietary intervention period, seasonality and small number of participants in the intervention groups. Further and more detailed investigation into markers of inflammation and increase in whole grain intake is required.

In common with some cross-sectional and observational studies, we were unable to see significant differences in blood pressure, BMI and blood analytes^(^ ^21^ ^,^ ^32^ ^,^ ^33^ ^)^, including cholesterol concentrations between consumers and non-consumers and across levels of intake^(^ ^34^ ^)^. This may be due to the overall low whole grain intake seen in this population, and also the relatively small sample size. Fewer than 30 % of the population achieved the minimum recommended whole grain intake that may reduce the risk of CVD, T2D, overweight and obesity^(^ ^3^ ^)^. With the majority of population consuming such low levels of whole grain, it will be difficult to detect any improvement in health markers.

### Conclusion

Whole grain intake for over 70 % of this population fell below dietary recommendation in other countries for decreased risk of CVD and T2D. This low intake of whole grain may explain the small variation across markers of health in this population. After adjustment for sex and age, only significant associations of increased whole grain intake with a fall in leucocyte counts and a significant difference in CRP concentrations between the low consumers and non-consumers (in adults only) were seen. The nutrient intakes of whole grain consumers compared with the non-consumers were closer to dietary reference values, particularly for fibre intake, suggesting that increasing whole grain intake is associated with improved diet quality. The results suggest that even small intakes of whole grain may contribute to better health and diet pattern, and that increased whole grain intake should be encouraged.
